# Significance of OCT3/4 and SOX2 antigens expression by leukemic blast cells in adult acute leukemia

**DOI:** 10.1186/s43046-024-00209-3

**Published:** 2024-02-12

**Authors:** Salah Aref, Omnyia Khaled, Nadia El Menshawy, Emad Azmy, Mohamed Aref, Osama Salama, Nada Khaled

**Affiliations:** 1 Hematology Unit, Mansoura University Oncology Center, Mansoura University, Mansoura, Egypt; 2 Hematology Unit, Internal Medicine Department, Faculty of Medicine, Mansoura University, Mansoura, Egypt; 3 Internal Medicine Department, Mansoura Faculty of Medicine, Mansoura University, Mansoura, Egypt

**Keywords:** AML, ALL, SOX2, OCT3/4, Outcome

## Abstract

**Objective:**

This study aimed to address the prognostic impact of SOX2 and OCT3/4 expression on adult acute leukemia patients’ outcomes.

**Methods:**

SOX2 and OCT3/4 expression by blast cells were evaluated by flow cytometry in 80 acute leukemia patients and 8 healthy controls.

**Results:**

Baseline SOX2 and OCT3/4 expression were significantly higher in both ALL (*P* = < 0.001, *P* = 0.005 respectively) and AML patients (*P* < 0.001, *P* = 0.003 respectively) as compared to control, and decline at complete remission (CR) and elevated again at relapse. High SOX2 and OCT3/4 levels were significantly correlated with the presence of adverse risk stratification parameters.

**Conclusion:**

Our findings indicated that both SOX2 and OCT3/4 could serve as biomarkers that could improve risk stratification of acute leukemia patients. Also, both SOX2 and OCT3/4 might be a therapeutic target, especially in resistant acute leukemia.

## Introduction

Acute leukemia (AL) is a clonal disease of hematopoietic stem cells in which uncontrolled cell expansion and differentiation occur. AL is classified according to the affected lineage into acute lymphocytic leukemia (ALL) and acute myeloid leukemia (AML) [1]. AML is one of the aggressive malignant hematological disorders all over the world, accounting for 80–90% of adult acute leukemia, but only accounts for 15–20% of children’s leukemia. In 2022, the estimated number of new cases in the USA was 20,050 and the estimated number of deaths was 11,540 [2]. The aggressiveness of the disease is reflected by the 5-year survival rate of 30.5% based on data collected in the period, 2012–2018 [3].

ALL is more frequently reported in children. Its incidence is bimodal with the first beak in childhood (80% of ALL) and the second beak around age 50 [4]. In 2022, the estimated number of new cases was 6660 and the estimated number of deaths was 1560 in the USA [2]. Although children with ALL have 90% 5-year overall survival (OS), only 25% of older patients (more than 50 years old) were alive 5 years after diagnosis, thus emphasizing the need for further improvements in treatment for older patients [5].

The higher frequency of relapse, which had a negative impact on OS [6] had been attributed to a fraction of the leukemic population that re-expresses embryonic cell markers as Oct-3/4 and Sox2, which have a role in the activation of genes responsible for self-renewal and suppression of genes involved in cell differentiation [7].

SOX/Sox (SRY homology box) protein family composed of 20 members in man and mouse [8], the most explored of them is SOX2/Sox2 [9]. SOX2 regulates several features of cancer as proliferation, migration, invasion, metastasis, tumor initiation, cancer stem cell formation as well as resistance to apoptosis and therapy through cross-reacting with a variety of signaling pathways [10]. It was reported that SOX2-positive cells remain as a small population of multipotent stem and/or progenitor cells in the adult pituitary gland [11].

Octamer-binding transcription factor 4 (OCT4, also known as OCT3 or OCT3/4) is a transcription factor that binds as an octamer. It was first discovered in 1989 [12], and its expression was observed in many sites as ovulated oocytes, early pre-implantation embryos, primitive ectoderm, the inner cell mass, ESCs, embryonic germ cells, and embryonic carcinoma cells [13, 14]. OCT3/4 acts as a transcription factors that contribute to carcinogenesis, tumor metastasis, and poor results [15]. It has been reported that OCT3/4 may be a therapeutic target, because the downregulation of OCT3/4 in cells leads to the loss of self-renewal and proliferation abilities, favoring the process of apoptosis in cancer cells [16]. Few previous studies have evaluated the expression of SOX2 and OCT3/4 expression in AML patients; however, no one study evaluated both antigens in adult ALL patients.

The aim of our study was to assess the impact of bone marrow blast cells SOX2 and OCT3/4 expression by blast cells on acute leukemia patients’ outcomes.

## Material and methods

### Subjects

This study included 80 newly diagnosed patients with acute leukemia (42 AML, 38 ALL) (28 females and 52 males) their median age is 39 years (age range 18 to 81 years); attending the outpatient clinic of our center during the period between January 2019 and January 2023. The follow-up was at first every few weeks and then every 3 months for 2 years. The bone marrow (BM) samples were collected from 8 healthy controls (BM donors) of matched age and gender. The study protocol was reviewed and approved by the Institutional Review Board [MD.18.03.19.R1]. All patients participating in this study gave written informed consent according to the declaration of Helsinki.

### Inclusion criteria

Newly diagnosed adult acute leukemia patients.

### Exclusion criteria

Leukemic patients younger than 18 years; acute leukemia combined with other malignancies acute leukemia under therapy; secondary acute leukemia.

All patients’ clinical data, age, gender, and full hemogram were collected from patients’ medical records The diagnosis was based on FAB and 2016 WHO classification and confirmed by immunophenotyping using a mixture of monoclonal antibodies panel for acute leukemia and cytogenetic studies by FISH technique. A total number of 42 patients were classified as AML and 38 patients were classified as ALL. FAB subtypes of AML patients included M1-M2 (45.2%), M3 (9.5%), M4-5 (42.9%), and M7 (2.4%). ALL patients were categorized into B ALL (78.9%) and T ALL (21.1%). The patients were followed up for 24 months. During the follow-up period, we recorded that 59 patients achieved complete remission after induction treatment, and 19 patients developed relapse.

AML patients were treated with a cytarabine-based intensive chemotherapy regimen with different dosages during induction therapy based on performance status, while ALL patients were treated with pediatric-inspired protocol plus tyrosine kinase inhibitors (only for Philadelphia chromosome-positive cases) and hyper-CVAD for adult patients. Relapsed/refractory cases were treated by either HAM (high-dose cytarabine and mitoxantrone) or FLAG (fludarabine, cytarabine, and G-CSF) protocol [17].

Response to treatment was categorized into 4 groups: *complete response* (CR); no circulating blasts or extramedullary disease, trilineage hematopoiesis (TLH) and < 5% blasts, absolute neutrophil count (ANC) > 1000/μL, platelets > 100,000/μL and no recurrence for 4 weeks. *Refractory disease* was a failure to achieve CR at the end of induction. Induction death (ID) was defined as the occurrence of death during the induction phase. *Hematological relapse* was defined as a reappearance of blasts in the blood or bone marrow (> 5%) or in any extramedullary site after a CR. *MRD detection* was carried out at the following points: day 28 post-induction, every 3 months in the first year, and every 6 months in the 2nd year. Complete remission was defined by the presence of negative MRD [18].

## Methods

### Blast cells expression of SOX2 and OCT3/4 antigens were identified by flow cytometry

Bone marrow EDTA samples were processed within 2 h from sampling. Fresh bone marrow samples were processed as follows: 100 μl of the sample was added to 100 µl of cold Fixation Buffer (R1) and the mixture was vortexed. Blank and sample tubes were incubated at room temperature for 15 min in the dark, then washed with 2 ml of phosphate-buffered saline (PBS), after the tubes were centrifuged at (1250–1500 rpm/350–500×*g* for 5 min) and the supernatant was decanted. One hundred microliters of Permeabilization buffer (R2) were added and incubated at room temperature for 5 min in the dark. Then, 10 µL of conjugated antibody SOX2 PE and OCT3/4 APC (R&D system, Inc., Minneapolis, MN, USA) were added and vortexed. Tubes were incubated for 15 min at room temperature in the dark. Two times washing was done using 2 ml of PBS. The cells were suspended in 300 µL PBS buffer for flow cytometric analysis. Acquisition of a minimum of 50,000 events was done employing (Navios EX Flow Cytometer (Beckman Coulter).

**Fig. 1:**
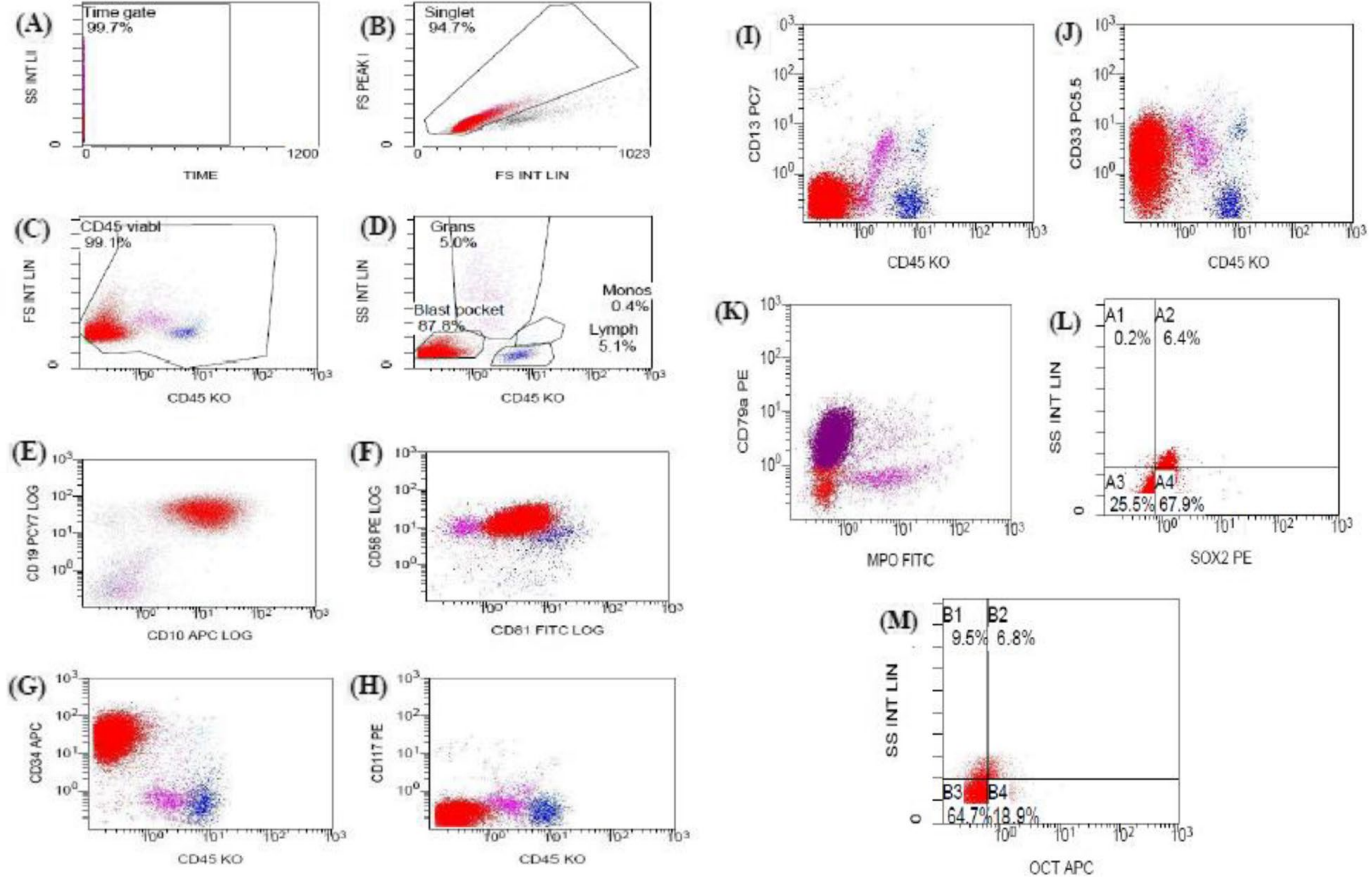
Immunophenotyping of ALL case.** A** Time versus SS to ensure continuity of cell flow. **B** FS peak versus FS integral to include only singlet cells. **C** CD45 versus FS to exclude debris and dead cells. **D** CD45 versus SS to gate blast cells in low SS and dim CD45. **E** Positive CD19, CD10 expression on blast cells. **F** Positive CD58, CD81 expression on blast cells. **G** Positive CD34 expression on blast cells.** H** Negative CD117 expression on blast cells.** I** Negative CD13 expression on blast cells. **J** Aberrant CD33 expression on blast cells. **K** Positive CD79a expression on blast cells.** L** SOX2 versus SS to calculate the expression of SOX2 as a percent of blast cells in the A4 compartment. **M** OCT3/4 versus SS to calculate expression of OCT3/4 as percent of blast cells in B4 compartment

**Fig. 2:**
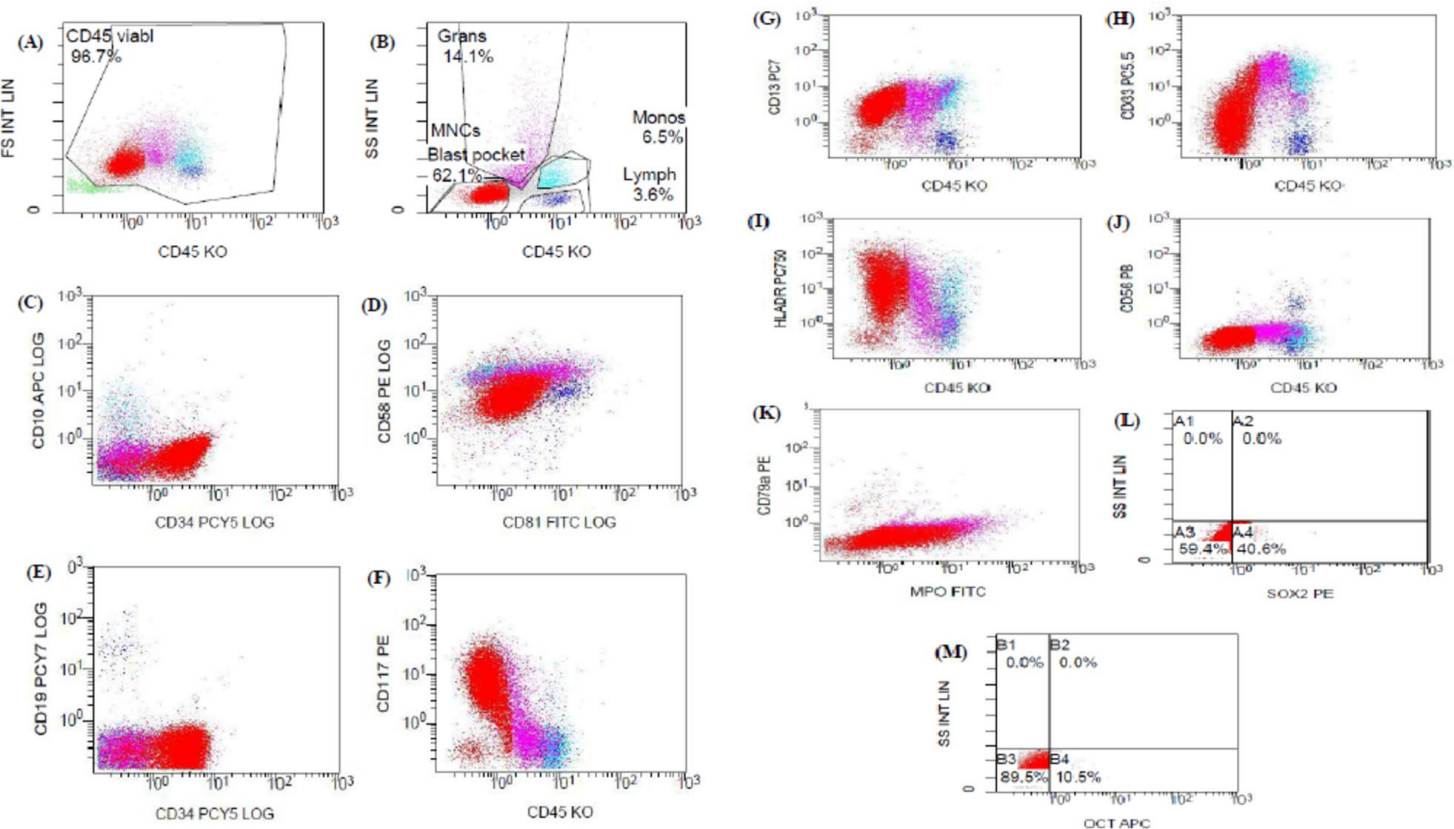
Immunophenotyping of AML case. **A** CD45 versus FS to exclude debris and dead cells. **B** CD45 versus SS to gate blast cells in low SS and dim CD45.** C** Positive CD34 and negative CD10 expression on blast cells. **D** Positive CD58, CD81 expression on blast cells. **E** Positive CD34 and negative CD19 expression on blast cells. **F** Positive CD117 expression on blast cells.** G** Positive CD13 expression on blast cells. **H** Positive CD33 expression on blast cells. **I** Positive HLADR expression on blast cells.** J** Negative CD56 expression on blast.** K** Positive MPO and negative CD79a expression on blast cells. **L** SOX2 versus SS to calculate the expression of SOX2 as a percent of blast cells in the A4 compartment. **M** OCT3/4 versus SS to calculate expression of OCT3/4 as a percent of blast cells in the B4 compartment

Initial gating was done using forward scatter area on *X*-axis versus forward scatter height on the *Y*-axis to include only singlet cells, then another gate was done using CD45 versus forward scatter (FSC) to exclude debris and dead cells. Then blast cell population were gated as low side scatter (SCC) in dim CD45, confirmed by expression of immature markers such as (CD34, CD117, HLA-DR, and CD99) that distinguished blast cells from maturing cells and calculated as a percentage of the total number of gated events. Assignment of blast lineage was assessed by different markers evaluated (CD34, CD117, HLA-DR, CD13, CD33, MPO, CD64, CD14, CD36, CD11b) for AML cases and CD19, CD10, CD20, cytoplasmic CD22, cytoplasmic CD79a, CD38, CD81, CD58, CD56 for B-ALL cases and cytoplasmic CD3, surface CD3, CD2, CD4, CD5, CD7, CD8, CD1a and CD99 for T-ALL cases (Table 1). Expression of SOX2 and OCT3/4 was assessed as a percentage of the blast cells in the plot displayed marker on *X*-axis versus side scatter (SSC) on *Y*-axis (Figs. 1 and 2).

**Table 1:** The flour chrome monoclonal antibodies and their source used in our study

Antibodies	Supplier	Clone	Fluorochrome
Mouse anti-CD45	Beckman Coulter	J33	ECD
Mouse anti-CD34	Beckman Coulter	581	FITC
Mouse anti-CD117	Beckman Coulter	104D2D1	PE
Mouse anti-HLA-DR	Beckman Coulter	Immu-357	APC-A750
Mouse anti-CD13	Beckman Coulter	Immul03.44	PC7
Mouse anti-CD33	Beckman Coulter	D3HL60.251	PerCP/Cy5.5
MPO	Beckman Coulter	CLB-MPO-01	FITC
Mouse anti-CD64	Beckman Coulter	22	PE
Mouse anti-CD14	Beckman Coulter	RM052	APC-A750
Mouse anti-CD36	Beckman Coulter	FA6.152	FITC
Mouse anti-CD11b	Beckman Coulter	Bear1	PerCP/Cy5.5
Mouse anti-CD19	Beckman Coulter	J3-119	PC7
Mouse anti-CD10	Beckman Coulter	ALB1	APC
Mouse anti-CD20	Beckman Coulter	B9E9	APC-A750
Mouse anti-CD22	Beckman Coulter	SJ10.1H11	PerCP/Cy5.5
Mouse anti-CD79a	Beckman Coulter	HM47	PE
Mouse anti-CD38	Beckman Coulter	LS198-4.3	Pacific Blue
Mouse anti-CD81	Beckman Coulter	JS64	FITC
Mouse anti-CD58	Beckman Coulter	ALCD58	PE
Mouse anti-CD56	Beckman Coulter	N901	PC7
Mouse anti-CD3	Beckman Coulter	UCHT1	PC7
Mouse anti-CD2	Beckman Coulter	39C1.5	APC-A750
Mouse anti-CD4	Beckman Coulter	SFCl12T4D11	PerCP/Cy5.5
Mouse anti-CD5	Beckman Coulter	BL1a	Pacific Blue
Mouse anti-CD7	Beckman Coulter	8H8.1	FITC
Mouse anti-CD8	Beckman Coulter	B9.11	APC-A750
Mouse anti-CD1a	Beckman Coulter	BL6	APC
Mouse anti-CD99	Beckman Coulter	3B2/TA8	PE
SOX2	R&D system	245610	PE
OCT3/4	R&D system	240408	APC

The expression for OCT3/4 and SOX2 in normal lymphocytes gated in normal bone marrow samples was considered the cutoff level. The patients harboring expression levels above this cutoff were considered positive.

Isotype control (antimouse IgG1FITC/Ig2αPE) is used as a negative control for assessing blast cell expression to determine the degree of positivity of leukemic panels.

### Normal bone marrow

Staining of bone marrow cells by a combined mixture of CD34/OCT3/4, or CD34/SOX2 to address and quantify CD34+ population expressing OCT3/4 and SOX2. Staining of the lymphocytes by OCT3/4 and SOX2 was used as a negative control [19].

### Statistical analysis

Data were analyzed using the Statistical Package of Social Science (SPSS) program for Windows (Standard version 21). The normality of data was first tested with a one-sample Kolmogorov-Smirnov test. Qualitative data were described using numbers and percentages. Continuous variables were presented as median (min–max) for non-normal data. For comparing two groups Mann-Whitney test (non-parametric) was used and for more than two groups Kruskal-Wallis test was used. Wilcoxon test was used for comparing non-parametric data in one group. The ROC curve (receiver operating characteristic) was used to assess the sensitivity and specificity for quantitative diagnostic measures that categorize cases into one of two groups. AUC (area under the curve) is the area between the curve and the reference line, it represents how the model is capable of distinguishing between classes. Kaplan-Meier test was used for survival analysis and the statistical significance of differences among curves was determined by log-rank test. Cox’s proportional hazards regression models were used to evaluate factors affecting DFS and OS. For all the above-mentioned statistical tests done, the threshold of significance is fixed at a 5% level (*p* value). The results were considered significant when the *p* ≤ 0.05. The smaller the *p* value obtained, the more significant the results. The sample size was calculated using an online sample size calculator (https:// https://riskcalc.org/samplesize/) with an estimated mean of OCT-4 expression [20] and level of alpha error of 5% and study power of 95%. A minimal sample size required for the study is calculated to be 48 subjects. To account for possible drop out a total sample of 80 subjects in each group is initially planned to be included in the study.

## Results

### SOX2 and OCT3/4 expression in the studied patient

#### Levels of expression at diagnosis

Both ALL and AML groups had higher levels of SOX2 expression as compared to control (*P* < 0.001 for both) and OCT3/4 expression (*P* = 0.005 and *P* = 0.003 respectively) as compared to controls. No significant difference between ALL and AML patient groups was found with regard to SOX2 and OCT3/4 levels (*P* = 0.128, *P* = 0.832 respectively) (Table 2).

**Table 2:** Comparison of SOX2 and OCT3/4 expression levels at diagnosis among studied groups

Parameter	Control (*N* = 8)	ALL (*N* = 38)	AML (*N* = 42)	*P*^*1*^	*P*^*2*^	*P*^*3*^	*P*^*4*^
SOX2 expression (%)	0.51% (0.25–1.13)	16.3% (0.3–71.6)	25.7% (0.1–81.8)	< 0.001	< 0.001	0.128	< 0.001
OCT3/4 expression (%)	0.45 (0.13–0.81)	1.35 (0.1–35.3)	1.7 (0.1–14.0)	0.005	0.003	0.832	0.013

Continuous variables are expressed as median (min–max). Data are compared using Kruskal-Wallis, Mann-Whitney tests. *P*^*1*^ between control and ALL group. *P*^*2*^ between the control and AML groups. *P*^*3*^ between ALL and AML groups. *P*^*4*^ between 3 groups

### Levels of SOX2 and OCT3/ expression at remission

**Fig. 3:**
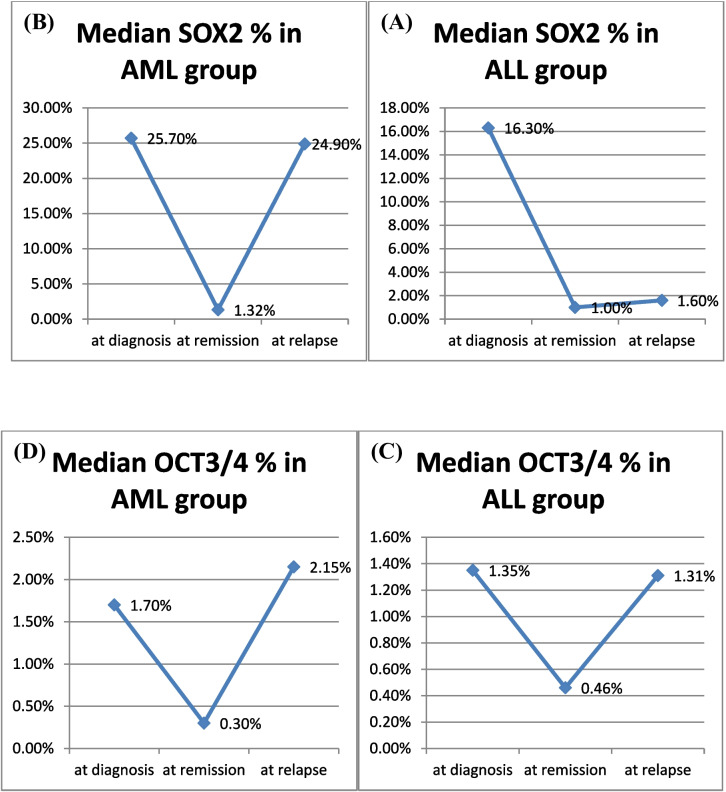
Kinetic expression of SOX2 in ALL and AML patient groups (**A**, **B**) and OCT3/4 in ALL and AML patient groups (**C**, **D**). Both ALL and AML patients achieved CR showed significantly lower levels of SOX2 and OCT3/4 compared to levels detected at diagnosis. In relapsed cases, levels of SOX2 and OCT3/4 were significantly higher than levels at CR in both ALL and AML cases

At bone marrow remission the expression levels of both SOX2 and OCT3/4 were significantly downregulated as compared to diagnosis levels and the differences were statistically significant in AML [for SOX2; *P* = < 0.001 and for OCT3/4; *P* = < 0.001] and in ALL group [for SOX2; *P* = < 0.001 and for OCT3/4; *P* = < 0.001] (Tables 3 and 4) (Fig. 3).

**Table 3:** Comparison of SOX2 expression at diagnosis, remission, and relapse among leukemic patients

Parameter	ALL (*n* = 38)	AML (*n* =4 2 )
	Median (min–max)	Median (min–max)
SOX2 expression (%) at diagnosis	16.3% (0.3–71.6)	25.7% (0.1–81.8)
SOX2 expression (%) at remission	1.0% (0.20–9.16)	1.32% (0.03–14.0)
SOX2 expression (%) at relapse	1.6 % (0.3–67.8)	24.9 % (2.61–87.0)
*P*^*1*^	< 0.001	< 0.001
*P*^*2*^	0.066	0.386
*P*^*3*^	0.008	0.005

Wilcoxon test, *P*^*1*^ between SOX2 level at diagnosis and remission in each group, *P*^*2*^ between SOX2 level at diagnosis and at relapse in each group,* P*^*3*^ between SOX2 level at remission and at relapse in each group

**Table 4:** Comparison of OCT3/4 expression at diagnosis, remission, and relapse among leukemic patients

Parameter	ALL (*n* = 38)	AML (*n* = 42)
	Median (min–max)	Median (min–max)
OCT3/4 % at diagnosis	1.35 (0.1–35.3)	1.7 (0.1–14.0)
OCT3/4 % at remission	0.46 (0.02–2.1)	0.30 (0.02–1.0)
OCT3/4 %at relapse	1.31 (0.3–10.5)	2.15 (1.4-49.6)
P^1^	< 0.001	< 0.001
P^2^	0.515	0.475
P^3^	0.008	0.005

Wilcoxon test, *P*^*1*^ between OCT3/4 level at diagnosis and remission in each group, *P*^*2*^ between OCT3/4 level at diagnosis and at relapse in each group, *P*^*3*^ between OCT3/4 level at remission and at relapse in each group

### Levels of SOX2 and OCT3/4 expression at relapse

In the AML group; both SOX2 and OCT3/4 were elevated in relapse compared to their level at remission (*P* = 0.005, *P* = 0.005 respectively), also in the ALL group, relapsed cases had significantly higher SOX2 and OCT3/4 levels compared to remission (*P* = 0.008, *P* = 0.008 respectively) (Tables 3 and 4) (Fig. 3).

### Impact of OCT3/4 and SOX2 expressions on acute leukemia patients’ outcome

**Fig. 4:**
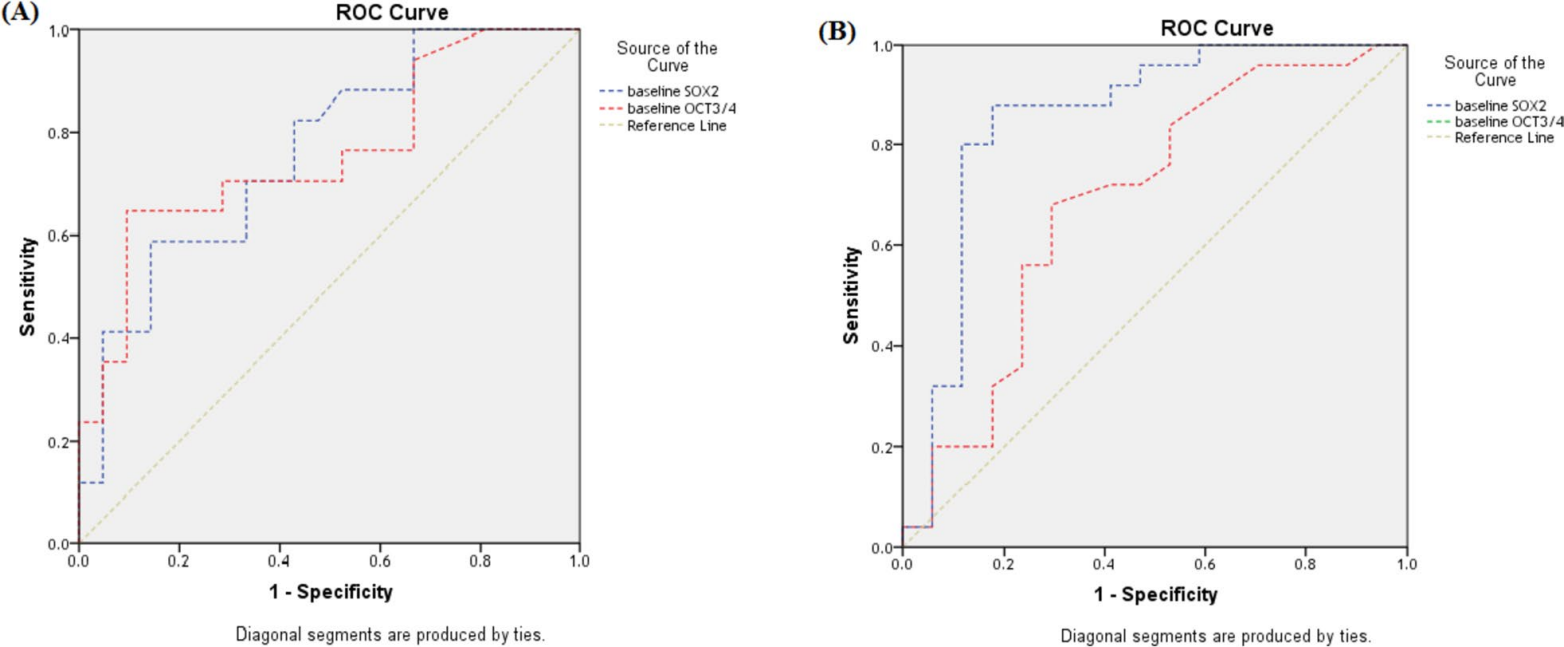
ROC curve (**A**) to identify the optimal SOX2 and OCT3/4 expression cutoff value that predicts death in ALL (best cut-off for SOX2 > 16.3% with AUC 0.763, *P* = 0.006, sensitivity 70.6% and specificity 66.7%) and (best cut-off for OCT3/4 > 1.55% with AUC of 0.758, *P* = 0.007, sensitivity 70.6% and specificity 71.4%). **B** To identify the optimal SOX2 and OCT3/4 expression cutoff value that predicts death in AML (best cut-off for SOX2 > 21.7% with AUC 0.854, *P* = < 0.001, sensitivity 88.0% and specificity 82.4%) and (best cut-off for OCT3/4 > 1.65% with AUC of 0.692, *P* = 0.037, sensitivity 68.0% and specificity 70.6%)

ROC curve was used to evaluate the cut-off value of OCT3/4 and SOX2 as prognostic markers for survival in both ALL and AML. In ALL, ROC curve analysis demonstrated that SOX2 ≥ 16.3% and OCT3/4 ≥ 1.55% identified patients likely to experience death with AUC (area under the curve) of 0.763 (*P* value = 0.006) sensitivity 70.6% and specificity 66.7% and AUC of 0.758, (*P* value = 0.007), sensitivity 70.6% and specificity 71.4% respectively (Fig. 4A).

In AML, SOX2 ≥ 21.7% and OCT3/4 ≥ 1.65% identified patients likely to experience death with AUC of 0.854 (*P* value = < 0.001), sensitivity 88.0% and specificity 82.4% and AUC of 0.692 (*P* value = 0.037), sensitivity 68.0% and specificity 70.6% respectively (Fig. 4B).

### Evaluation of OCT3/4 and SOX2 expression in relation to clinicopathological features

In ALL cases; both SOX2 and OCT3/4 markers were significantly elevated in unfavorable cytogenetic risk stratification compared to favorable risk stratification, in non-CR cases compared to CR cases, in relapsed cases compared to non-relapsed cases, and in dead patients compared to living patients. SOX2 was significantly elevated in T-ALL cases compared to B ALL cases (Table 5).

**Table 5:** Association of SOX2 and OCT3/4 expression with demographic, clinical, and laboratory data of ALL patients

Parameter		SOX2 expression (%) Median (min–max)	OCT3/4 expression (%) Median (min–max)	*P*^*1*^	*P*^*2*^
Gender	Male	13.0 (0.3–67.9)	1.2 (0.1–35.3)	0.174	0.174
	Female	25.6 (3.1–71.6)	2.1 (0.2–6.7)		
CD34%	Negative	8.3 (0.3–46.2)	0.9 (0.1–35.3)	0.101	0.194
	Positive	20.2 (1.3–71.6)	1.7 (0.1–19.5)		
Immunological subtype	B ALL	13.0 (0.3–71.6)	1.3 (0.1–19.5)	0.034	0.792
	T ALL	39.0 (7.1–54.0)	1.7 (0.3–35.3)		
Risk stratification	Favorable	7.0 (0.3–34.5)	1.0 (0.1–3.1)	<0.001	0.006
	Unfavorable	32.3 (6.6–71.6)	2.5 (0.2–35.3)		
Response	CR	10.8 (0.3–67.9)	1.2 (0.1–13.2)	0.017	0.038
	Non-CR	37.2 (6.6–71.6)	3.3 (0.3–35.3)		
Relapse	Non relapsed	9.7 (0.3–44.3)	1.1 (0.1–3.6)	0.019	0.049
	Relapsed	29.8 (7.1–67.9)	1.9 (0.3–13.2)		
Living status	Lived	9.6 (0.3–54.0)	1.1 (0.1–6.6)	0.005	0.006
	Dead	32.2 (5.8–71.6)	2.8 (0.3–35.3)		

Mann-Whitney test. *P*^*1*^ between groups as regards SOX2 level, *P*^*2*^ between groups as regards OCT3/4 level

In AML cases, poor and intermediate cytogenetic risk stratification had higher SOX2 and OCT3/4 levels compared to favorable risk stratification, also in non-CR patients compared to patients achieved CR, in relapsed cases compared to non-relapsed cases, and in dead patients compared to living patients (Table 6).

**Table 6:** Association of SOX2 and OCT3/4 expression with demographic, clinical, and laboratory data of AML patients

Parameter		SOX2 expression (%)Median (min–max)	OCT3/4 expression( %)Median (min–max)	*P*^*1*^	*P*^*2*^
Gender	Male	25.5 (0.6–81.8)	1.7 (0.2–14.0)	0.877	0.795
	Female	25.8 (0.1–71.9)	1.6 (0.1–8.8)		
CD34%	Negative	14.9 (0.1–79.1)	1.2 (0.1–6.5)	0.066	0.133
	Positive	30.9 (1–81.8)	1.7 (0.2–14.0)		
FAB Subtypes	M1-M2	32.7 (1.0–71.9)	1.7 (0.5–10.7)	0.351	0.588
	M3	7.3 (0.1–78.9)	3.4 (0.1–14.0)		
	M4-M5	23.5 (0.6–81.8)	1.3 (0.2–8.8)		
Risk stratification	Favorable	11.5 (0.1–26.9)	0.5 (0.1–5.3)	< 0.001	0.003
	Intermediate	25.5 (9.6–79.1)	1.7 (0.4–6.5)		
	Poor	53.4 (24.7–81.8)	2.3 (0.5–14.0)		
Response	CR	16.7 (0.1–79.1)	1.34 (0.1–6.5)	< 0.001	0.006
	Non-CR	54.5 (24.7–81.8)	2.3 (0.5–14.0)		
Relapse	Non relapsed	12.7 (0.1–65.0)	0.5 (0.1–6.5)	0.045	< 0.001
	Relapsed	24.5 (6.9–79.1)	2.0 (0.8–5.7)		
Living status	Lived	11.5 (0.1–79.1)	0.7 (0.1–5.7)	< 0.001	0.012
	Dead	35.1 (9.6–81.8)	1.9 (0.2–14.0)		

Mann-Whitney test, .Kruskal-Wallis *P*^*1*^ between groups as regards SOX2 level, *P*^*2*^ between groups as regards OCT3/4

### Impact of OCT3/4 and SOX2 expression on OS and DFS

Based on ROC curve analysis the acute leukemia patients were classified into 2 groups, ALL patients with high SOX2 expression (16.3%) and OCT3/4 (1.55%) expression. Also, AML patients were classified as those with high SOX2 expression and low SOX2 expression and also those with high OCT3/4 expression and low OCT3/4 expression according to ROC curve cut-off value of SOX2 (21.7%) and a cut-off value of OCT3/4 (1.65%) expression.

**Fig. 5:**
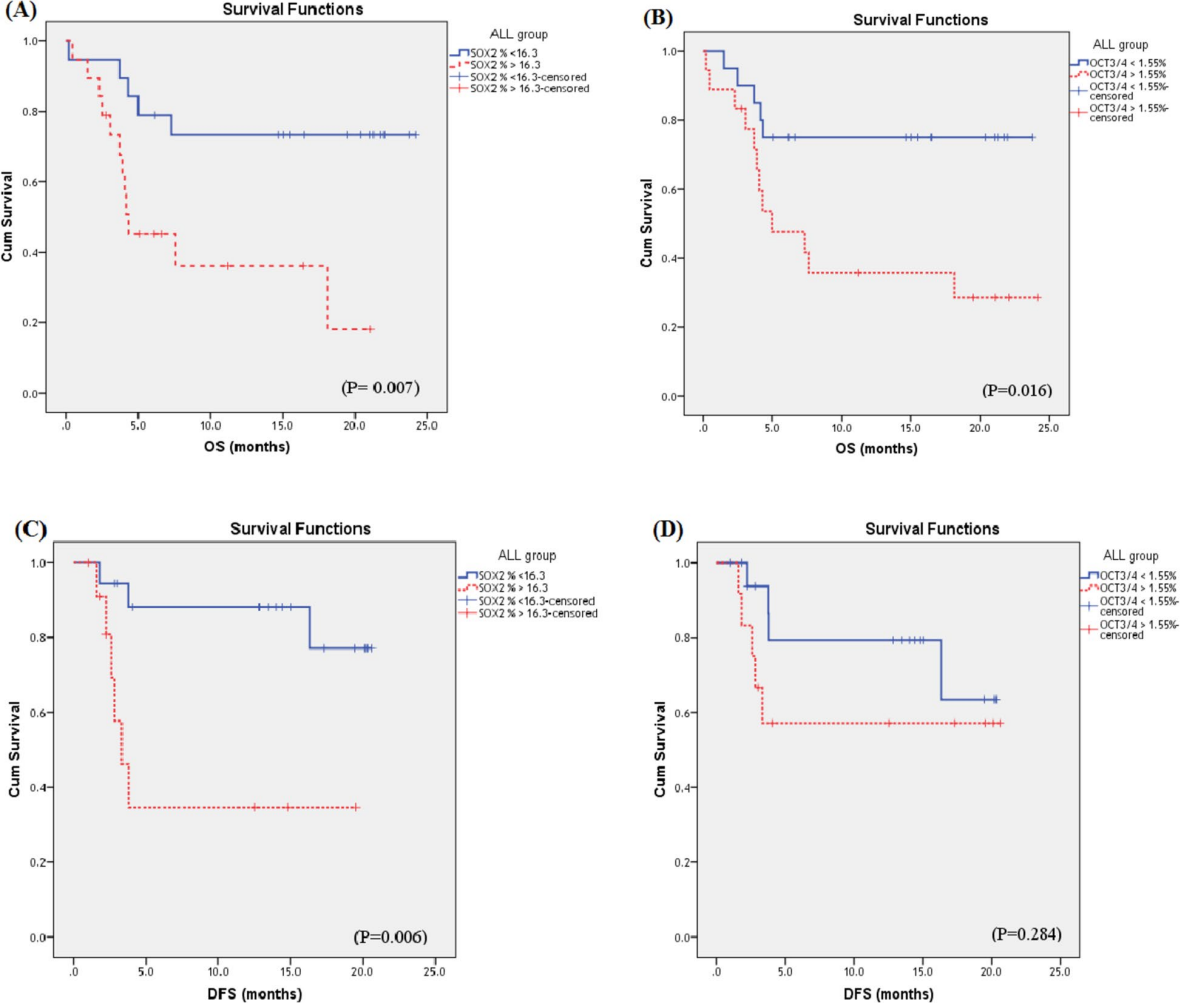
Kaplan-Meier curve for the impact of SOX2 expression and OCT3/4 expression on OS and DFS of ALL patients. **A**, **B** Patients with low SOX2 expression (< 16.3%) and low OCT3/4 expression (< 1.55%) had longer cumulative OS survival as compared to those with high expression (*P* = 0.007, and *P* = 0.016 respectively). **C**, **D** Patients with low SOX2 expression (< 16.3%) had longer cumulative DFS as compared to those with high expression (*P* = 0.006). While DFS was not significantly different between those with high and low OCT3/4 (*P* = 0.284)

**Fig. 6:**
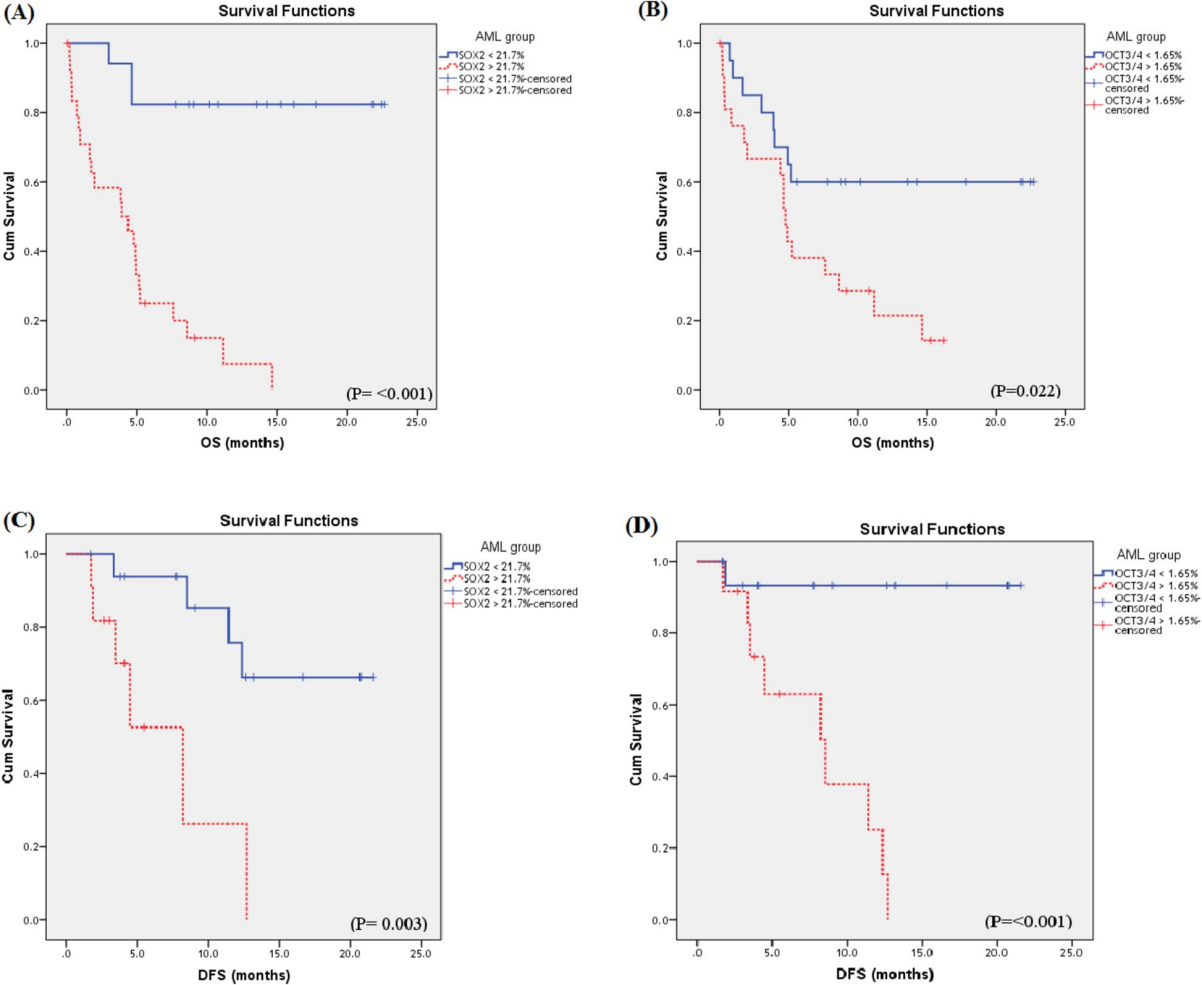
Kaplan-Meier curve for the impact of SOX2 expression and OCT3/4 expression on OS and DFS of AML patients. **A**, **B** Patients with low SOX2 expression (< 21.7%) and low OCT3/4 expression (< 1.55%) had longer cumulative OS survival as compared to those with high expression (*P* < 0.001), and (*P* = 0.022) respectively). **C**, **D** Patients with low SOX2 expression (< 21.7%) and OCTA3/4 had longer cumulative DFS as compared to those with high expression (*P* = 0.003, *P* = < 0.001 respectively)

Adult ALL patients expressed high SOX2 and OCT3/4 had significantly shorter OS and DFS as compared to those with low expression (*P* = 0.007; *P* = 0.016 respectively and *P* = 0.006 for SOX2) (Fig. 5). Moreover, AML, patients expressing high SOX2 and OCT3/4 had significantly shorter OS as compared to those with low SOX2 (*P* = < 0.001) and OCT3/4 expression (*P* = 0.022). As regards DFS, those with high SOX2 and OCT3/4 also had significantly shorter DFS than those with low SOX2 (*P* = 0.003) and OCT3/4 (< 0.001) (Fig. 6).

### COX regression analysis to identify biological factors that could predict CR, OS, and DFS

COX regression analysis was conducted to predict factor (s) affecting OS and DFS using age, gender, laboratory data, type, risk stratification, response, SOX2, and OCT3/4 as covariates. In ALL cases; non-CR (*P* = 0.021), high SOX2 expression (*P* = 0.029) and high OCT3/4 expression (*P* = 0.018) were significant independent factors for shorter OS in ALL patients in multivariate analysis. Moreover, both high SOX2 expression (*P* = 0.001) and high OCT3/4 expression (*P* = 0.025) were significant independent factors for shorter DFS in ALL patients in multivariate analysis (Tables 7 and 8).

**Table 7:** Cox regression analysis to predict the hazardous factor that could predict OS in ALL patients

Parameter	Univariable			Multivariable		
	*P*	HR	95% CI	*P* value	HR	95% CI
Age (years)	0.022	1.035	1.005–1.066	0.146	1.031	0.989–1.074
Gender	0.439	1.486	0.545–4.054			
WBCs × 10^9^/L	0.227	1.133	0.925–1.387			
Hemoglobin g/dl	0.674	0.998	0.990–1.007			
Platelets count × 10^9^/L	0.206	0.985	0.961–1.009			
BM blast cells %	0.118	1.006	0.989–1.011			
Risk stratification (unfavorable vs favorable)	0.011	7.926	2.234–28.125	0.120	3.703	0.710–19.299
Response (non-CR vs CR)	< 0.001	15.442	4.792–49.773	0.021	5.432	1.286–22.955
Relapse (relapsed vs non-relapsed)	0.522	1.585	0.387–6.490			
SOX2 expression (%)	0.001	1.041	1.017–1.066	0.029	1.029	1.003–1.056
OCT3/4 expression (%)	0.004	1.070	1.022–1.121	0.018	1.084	1.014–1.158

*HR* Hazard ratio, *CI* Confidence interval, (*r*) Reference group

**Table 8:** Cox regression analysis to predict the hazardous factor that could predict DFS in ALL patients

Parameter	Univariable			Multivariable		
	*P*	HR	95% CI	*P* value	HR	95% CI
Age (years)	0.419	1.015	0.979–1.053			
Gender	0.522	1.580	0.389–6.407			
WBCs × 10^9^/L	0.308	1.004	0.996–1.011			
Hemoglobin g/dl	0.108	0.983	0.949–1.095			
Platelets count × 10^9^/L	0.092	0.970	0.935–1.005			
BM blast cells %	0.084	1.007	0.999–1.015			
Cytogenetic risk stratification	0.045	4.014	1.031–15.630	0.664	1.471	0.258–8.404
SOX2 expression (%)	< 0.001	1.085	1.038–1.134	0.001	1.095	1.037–1.156
OCT3/4 expression (%)	0.004	1.660	1.179–2.338	0.025	1.575	1.059–2.343

*HR* Hazard ratio, *CI* Confidence interval, (*r*): Reference group

However, in AML cases; high SOX2 expression (*P* = 0.001) and high OCT3/4 expression (*P* = 0.045) were significant independent factors for shorter OS in AML patients in multivariate analysis. Moreover, both high SOX2 expression (*P* = 0.010) and high OCT3/4 expression (*P* = 0.023) were significant independent factors for shorter DFS in AML patients in multivariate analysis (Tables 9 and 10).

**Table 9:** Cox regression analysis to predict the hazardous factor that could predict OS in AML patients

Parameter	Univariable			Multivariable		
	*P*	HR	95% CI	*P* value	HR	95% CI
Age (years)	0.785	1.003	0.984–1.022			
Gender	0.784	1.117	0.506–2.464			
WBCs × 10^9^/L	0.827	1.001	0.955–1.006			
Hemoglobin g/dl	0.657	0.952	0.767–1.182			
Platelets count × 10^9^/L	0.367	0.996	0.989–1.004			
Bone marrow blast %	0.218	1.003	0.989–1.008			
Risk stratification						
Favorable	R	1	–	R	1	–
Intermediate	0.027	4.535	1.190–17.287	0.118	3.200	0.744–13.760
Poor	< 0.001	24.463	6.283–95.244	0.187	4.425	0.982–40.220
Response (non-CR vs CR)	< 0.001	10.294	4.107–25.802	0.052	6.078	0.982–37.634
Relapse (relapsed vs non-relapsed)	0.471	1.494	0.502–4.450			
SOX2 expression (%)	< 0.001	1.034	1.019–1.050	0.001	1.033	1.014–1.054
OCT3/4 expression (%)	0.001	1.179	1.027–1.353	0.045	1.163	1.021–1.339

*HR* Hazard ratio, *CI* Confidence interval, (*r*) Reference group

**Table 10:** Cox regression analysis to predict the hazardous factor that could predict DFS in AML patients

Parameter	Univariable			Multivariable		
	*P*	HR	95% CI	*P* value	HR	95% CI
Age (years)	0.651	1.012	0.993–1.024			
Gender	0.499	1.538	0.442–5.352			
WBCs × 10^9^/L	0.722	1.068	0.743–1.535			
Hemoglobin g/dl	0.513	0.996	0.984–1.008			
Platelets count × 10^9^/L	0.914	0.998	0.971–1.027			
BM blast cells %	0.690	1.002	0.993–1.011			
Risk stratification						
Favorable	*R*	1	–	*R*	1	–
Intermediate	0.020	5.104	1.290–20.195	0.163	3.086	0.633–15.046
Poor	0.035	4.135	1.173–15.127	0.219	2.726	0.578–10.060
SOX2 expression (%)	0.009	1.036	1.009–1.063	0.010	1.042	1.010–1.076
OCT3/4 expression (%)	0.016	1.442	1.045–1.992	0.023	1.543	1.061–2.245

*HR* Hazard ratio, *CI* Confidence interval, (*r*) Reference group

## Discussion

SOX2 and OCT3/4 act as transcription factors that confer stemness characteristics to the cancer cells which contribute to tumorigenesis, cancer metastasis, and poor prognosis [13]. SOX2 protein binds firstly to the chromosome and provides a target for subsequent OCT3/4 binding [21, 22]. This complex binds to specific target genes in the nucleus, induces their expression, and maintains embryonic characteristics [20].

In the current study, SOX2 and OCT3/4 expression were significantly higher in AML at diagnosis as compared to normal control. In patients who achieved CR, levels of SOX2 and OCT3/4 expression declined to the control levels. At relapse, both SOX2 and OCT3/4 expression elevated again to the diagnosis levels. These results are in agreement with the findings reported in previous studies [6, 23, 24]. In this context, similar findings were reported by Zhao et al. [20], and Picot et al. [19] who studied OCT3/4 expression by flowcytometry in BM samples harvested from 103 AML patients at diagnosis, and 40 samples at complete remission and 10 BM samples from healthy donors.

To our knowledge, no previous study had assessed the level of SOX2 expression in adult ALL. In our study, both SOX2 and OCT3/4 expression were significantly elevated at diagnosis as compared to control levels. At the remission phase, both markers decline to the control levels. At ALL relapse the expression levels of both markers elevated again. These findings indicate an association between the presence of blast cells and SOX2 and OCT3/4 expression. The role of embryonic markers in leukemogenesis is still under investigation. Both OCT4 and SOX2 are two of Yamanaka’s factors, which can induce pluripotency in differentiated cells to make stem cells with embryonic properties as they act as core transcription factors in maintaining pluripotency [25]. The forced expression of OCT3/4 transdifferentiates skin fibroblasts into hematopoietic cells without going through a stage of embryonic stem cells [26]. Meanwhile, OCT4 targets stem cell-specific growth factor genes, so the aberrant expression of OCT4 in leukemic cells may result in appropriate activation of stem cell growth factors that promote cell proliferation and decrease apoptosis.

ROC curve was used to determine the best cutoff value that could discriminate between high and low expression of SOX2 and OCT3/4. Acute leukemia patients with high SOX2 and OCT3/4 expression levels in the acute leukemia subgroup of patients were significantly associated with adverse risk stratification, non-CR patients, relapsed cases, and dead cases. In the same line, Yin et al. [23] demonstrated that over-expression of OCT4 in AML patients correlated with abnormal karyotypes and higher risk stratification. Xiang et al. [6] stated that higher OCT4 expression was linked to poor risk stratification and no relation with other clinicopathological features was detected. Tosic et al. [24] noted none of the patients with higher SOX2 levels were found in the favorable prognostic group. Picot et al. [19] reported that no correlation was detected between levels of any ESCA in leukemic cells with any of biological characteristics.

Both AML and ALL patients expressing higher levels of SOX2 and OCT3/4 groups have shorter OS and DSF as compared to patients with lower expression levels. These results go in harmony with Yin et al. [23] noted that patients with OCT4 high expression have significantly shorter overall survival (OS) as compared to those with lower expression. These findings suggest that high OCT3/4 expression has an adverse event and a worse impact on the prognosis of AML. Xiang et al. [8] classified AML patients by median of OCT4 expression into high and low groups, assessed survival and DFS and found that patients with low expression have better OS. Tosic et al. [24] stated that AML patients who have high SOX2 expression levels displayed shortened both OS and DFS compared to those with lower ones. In contrast, Picot et al. [19] stated that AML patients with higher SOX2 levels were associated with better OS but the difference was not statistically significant.

Cox regression analysis revealed that both SOX2 and OCT3/4 expression are independent predictors of shorter OS and DFS in both ALL and AML patients. In the same line, Xiang et al. [6] stated that OCT4 high expression was an independent predictor factor for worse OS and DFS in AML patients, it was explained as OCT4 interacts with many signaling pathways such as JAK/STAT pathway that accelerate proliferation, invasion of leukemic cells and aggravation of disease progress resulting in worse DFS and OS. However, no previous research studied this issue in ALL patients.

Several studies explained the role of high SOX2 expression in chemotherapeutic resistance that terminally leads to clinical relapse, These include (1) activation of multi-drug resistance ATP-binding cassette (ABC) transporter genes that lead to drug removal out of tumor cells such as in glioblastoma [26]) and breast cancer [27]. (2) Upregulation of antiapoptotic factor named B cell lymphoma 2 (BCL2) in lung cancer [28] and downregulation of proapoptotic protein NOXA [29]. (3) Activation of multiple signaling pathways mediating resistance to therapy, i.e., (a) WNT/β catenin signaling results in the development of tamoxifen resistance to in breast cancer [30] b) PI3K/Akt signaling that induces chemoresistance in glioma by enhancing ABC activity. Moreover, these mechanisms were proved in an experimental study done by Mukherjee et al. [27] who found that the knockdown of SOX2 increased chemosensitivity in breast cancer patients. Furthermore, the role of OCT3/4 overexpression in therapy resistance was declared by many studies [31–34] which mentioned the following mechanism: (1) OCT3/4 up-regulates miRNA-125 via interaction with its promoter, which acts as an oncogenic factor that inhibits apoptosis, (2) OCT3/4 regulates several signaling pathways as WNT/β catenin, JAK/STAT, and survivin/STAT3. OCT3/4 can regulate chemoradiation resistance and metastasis of hepatocellular adenocarcinoma through survivin/STAT3. Survivin plays a role in tumor progression by inhibition of apoptosis. OCT3/4 positively regulates survivin expression. (3) Higher expression of OCT3/4 enhances the activity of ABC in ovarian cancer that resists chemotherapy. Its role in drug resistance is proved by the knockdown of its expression, which increases sensitivity to chemotherapy and irradiation in lung cancer patients.

Both SOX2 and OCT3/4 could be a potential therapeutic target for the treatment of high-risk acute leukemia patients especially those who experienced resistance to chemotherapy. In this context, both markers could be used in the risk stratification of acute leukemia patients.

### Study limitations

Small number of normal bone marrow samples as well as a relatively small number of acute leukemia patients.

## Conclusion

Our findings indicated that both SOX2 and OCT3/4 could serve as biomarkers that could improve risk stratification of acute leukemia patients. Also, both SOX2 and OCT3/4 might be a therapeutic target, especially in resistant acute leukemia.

## Data Availability

The data that support the findings of this study are available from the corresponding author upon request.

## Abbreviations

AML: Acute myeloid leukemia
ALL: Acute lymphoblastic leukemia
BCL2: B cell lymphoma-2
CD: Cluster of differentiation
CR: Complete remission
DFS: Disease-free survival
OS: Overall survival
OCT4: Octamer-binding transcription factor 4
PD: Progressive course
PFS: Progression-free survival
PR: Partial remission
SOX/Sox: SRY homology box
